# Editorial: Graft preservation

**DOI:** 10.3389/fcvm.2024.1478730

**Published:** 2024-10-25

**Authors:** Dawn E. Bowles, Jamshid H. Karimov, Cristiano Amarelli

**Affiliations:** ^1^ Department of Surgery, Division of Surgical Sciences, Duke University Medical Center, Durham, NC, United States; ^2^ Department of Biomedical Engineering, Lerner Research Institute, Cleveland Clinic, Cleveland, OH, United States; ^3^ Cleveland Clinic Lerner College of Medicine, Case Western Reserve University, Cleveland, OH, United States; ^4^ Kaufman Center for Heart Failure, Heart, Vascular, and Thoracic Institute, Cleveland Clinic, Cleveland, OH, United States; ^5^ Department of Cardiac Surgery and Transplants, Monaldi, A.O. dei Colli, Naples, Italy

**Keywords:** machine perfusion (MP), cold storage (CS), heart transplant (HTx), organ reconditioning, organ banking, organ replacement regenerative therapy, gene therapy & therapeutic delivery

**Editorial on the Research Topic**
Graft preservation

Solid organ transplant outcomes have tremendously benefited from innovations in graft preservation strategies (1). *Ex-vivo* (or *ex-situ*) machine perfusion (EVMP) for organ preservation protects donor organs from the injuries traditionally encountered by cold static preservation (CSP), thus maintaining the organs in a “physiologic” state, minimizing rates of primary graft dysfunction (PGD) and promising functional recovery of marginal organs. EVMP has already demonstrated the capability to extend preservation periods well beyond what is considered acceptable after CSP for the kidney, liver, lung, and heart (2). EVMP strategies have expanded the donor pool and pushed the limits of the extended criteria toward donor organs previously excluded from transplantation (3). In this landscape, the use of donation after circulatory death (DCD) organs has been a game- changer that, before the emergence of the normothermic regional perfusion (NRP) technology (4), seemed strictly dependent on the availability of a platform to evaluate the organ *ex-situ*. The potential to recondition organs that could otherwise be discarded represents the “holy grail” of the new frontiers of EVMP (warm or cold). The challenge of extending preservation time and the growing use of EVMP during organ procurement have introduced the potential for administering therapeutics during this period to improve organ quality and mitigate post-transplantation complications. The already hot topic of heart allocation promises to be entirely revolutionized by the perspective of reducing the impact of ischemic time and broadening the area for the allocation, also encompassing immunologic compatibility, to a broader supranational geographic region (North America, Europe, Australia).

The primary objective of this research topic was to collect expert opinions, original research articles (clinical, translational, basic), case reports, and reviews (both brief and expansive) that address critical gaps in knowledge in the field of graft preservation and recent developments in applications of EVMP.

Lechiancole et al. set the stage for this collection by providing an extensive review of the use of various CSP and EVMP strategies in clinical heart transplants. The authors summarized the available clinical data and provided perspectives on technical aspects and limitations to current preservation techniques. Similarly, Iske et al. provided an interdisciplinary overview of the current abdominal and thoracic EVMP systems and organ-specific preservation protocols and summarized relevant EVMP applications beyond organ preservation for allogeneic transplantation.

Kasinpila et al. described an unusual case of a 55-year-old successfully retransplanted (21 years after the prior) with a DCD donor heart from a distant location, necessitating an extended transport period (>7 h) with normothermic EVMP. This article highlights the imbrication between perfusion technology and the expansion of DCD donation and shows how leading institutions are pushing the limits through EVMP.

There remains an unmet clinical need for a biomarker or a tool to ascertain organ quality during preservation. Mendiola Pla et al. applied video kinematic evaluation (Vi.Ki.E.) and assessed the feasibility of using this method to measure *ex vivo* cardiac kinematics. Porcine donor hearts underwent normothermic EVMP on the TransMedics® Organ Care System (OCS™). Vi.Ki.E. performed while the donor's hearts beat on the OCS™ could be applied to predict cardiac fitness and allow a reliable organ assessment.

Radomsky et al. compared the concentration levels of cytokines/chemokines in different perfusion solutions during *ex vivo* lung perfusion (EVLP) after 1 and 9 h of CSP using a porcine cardiac arrest model. While the concentrations of many inflammatory cytokines increased across all experimental groups, a longer period of CSP before EVLP did not result in an enhanced inflammatory protein secretion into perfusates. This knowledge may define the optimal lung preservation method that could potentially increase the donor lung pool.

A study by Niroomand et al. (from the Lund group in Sweden, which has driven the field of cytokine absorption in EVLP) utilized mass spectrometry-based proteomics and bioinformatics approaches to understand molecular mechanisms of how cytokine absorption impacts lung function when used during EVLP. This study revealed characteristic inflammatory, immunomodulatory, and coagulation pathway differences between the lungs treated with and without cytokine adsorption, which may lead to more targeted approaches to improve lung function.

Vervoorn et al.’s literature review focused on administering gene therapies delivered by EVMP. This review examined 23 studies of gene therapy applied to the heart during both hypothermic and normothermic EVMP conditions, using different vectors, perfusion conditions, duration of exposure to the vector, doses, and perfusion composition. Gene therapy delivered via EVMP has applications in both allo- and auto-cardiac transplantation. Autotransplantation during support with cardiopulmonary bypass may be the “moon-shot” to repair *ex-situ* a heart with a pathogenic mutation.

Ughetto et al. provide a comprehensive review of the mechanisms involved in ischemia- reperfusion injury to the heart during transplantation and existing targeted strategies useful to minimize injury leading to PGD. Treatments reviewed include pharmacological agents, gene therapy, cell therapy, metabolic modulation, and targeted drug delivery, all of which can be provided during EVMP. The most attractive solutions highlighted are blocking apoptosis and necrosis pathways, extracellular vesicle therapy, and donor heart-specific gene therapy.

McCully et al. provided an impressive overview of mitochondrial transplantation's potential as a novel methodology for rescuing cell viability and function following ischemia- reperfusion injury and its potential applications. The thoughtful amount of data supports the versatility and durability of such an approach, stimulating the interest in imbricating this technology with the current standard of graft preservation.

Andrijauskaite et al. utilized a newly developed portable hypothermic oxygenated machine perfusion device (the VP.S Encore) to evaluate unused human donor hearts. After placing these hearts on this innovative and simple-to-use cardiac preservation device for an extended period of time, the authors evaluated cardiac function by placing them in a Langendorff system for reperfusion and evaluation of cardiac contractility. These data constitute a step toward the clinical use of a device warranting hypothermic oxygenated perfusion. Provoost et al. report the first experience with the portable LUNGguard showing short-term outcomes that were safe and beneficial and the possibility of converting the transplant procedure to a diurnal activity. The opportunity to imbricate prolonged cold ischemic time and prolonged perfusion coupled with a reliable possibility of evaluating organ function offers further opportunities to redesign organ transplantation logistics.

Spencer et al. report efforts from the Extracorporeal Life Support Laboratory (University of Michigan) to prolong safe EVMP. Prior studies from this group demonstrated that the metabolic and biological basis for graft failure associated with prolonged EVMP was due to changes in blood composition over the perfusion period. In the current report, incorporating plasma exchange or ultrafiltration to the OCS circuits enabled successful perfusion of 24 h. In contrast, the majority of hearts perfused without these interventions failed between 10 and 21 h, with only one of these hearts lasting 24 h. The addition of intermittent left atrial (iLA) perfusion enabled real-time objective, quantifiable cardiac function assessment, a unique feature with a significant impact during the assessment of marginal and DCD donor hearts.

**Figure 1:**
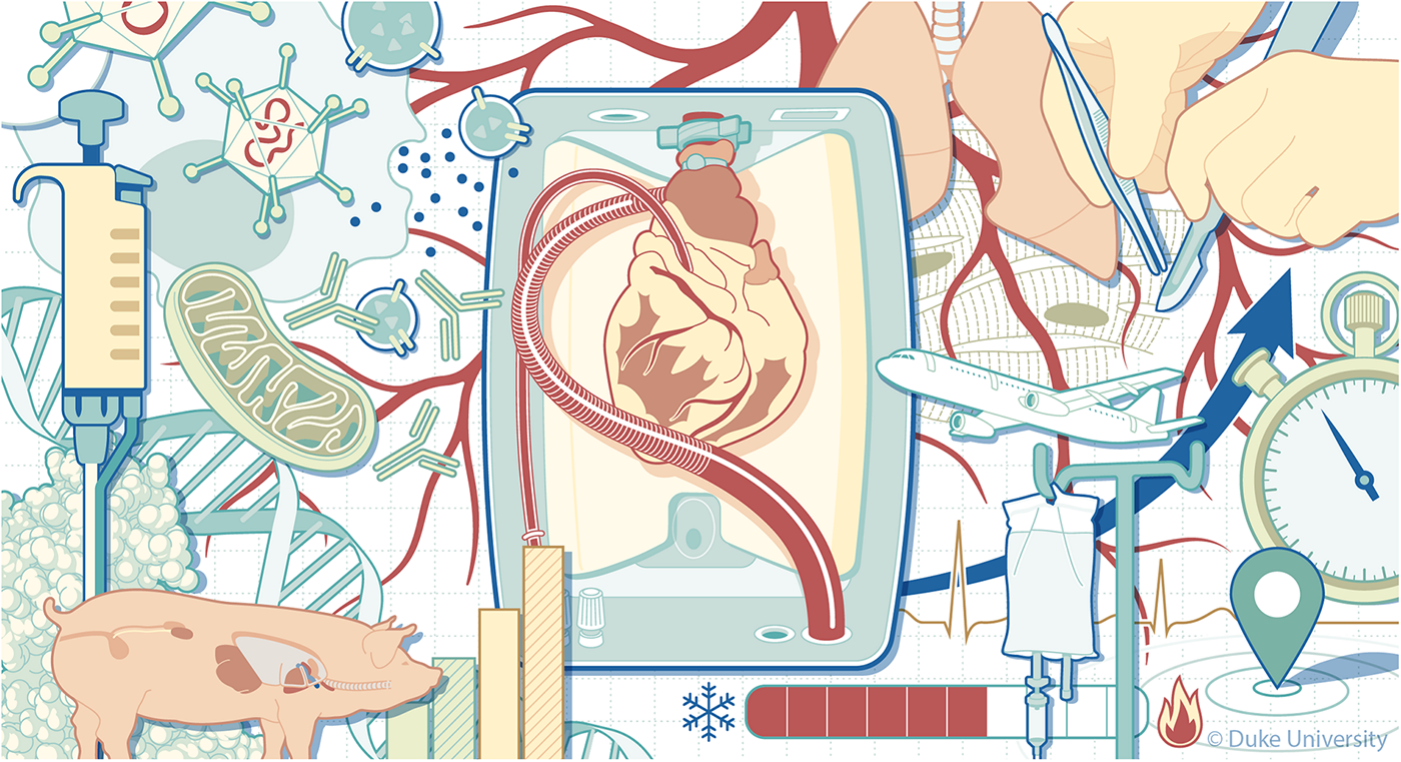
The challenges of extending machine perfusion length. Illustrated by Megan Llewellyn, MSMI (2024), copyright Duke University, licensed under CC BY-ND 4.0 with permission.

Overall, the underlying intention of this collection of articles was to emphasize the opportunity landscape for organ preservation techniques envisioned in Figure 1 and the betterment of perfusion outcomes, taking a closer look into cellular, molecular, and pathophysiological aspects of this continuously evolving area of research. The contributing papers provide extensive insight into clinical and engineering tools currently utilized to stimulate the development of better organ preservation techniques, supported by the most up-to-date research, device development, and clinical data. We hope that this issue serves as a foundation for further research in the areas that some of these articles have identified as lacking scientific information. These scientific gaps form the basis for identifying devices that can support prolonged graft perfusion and for moving from the current hurried logistical organization of transplant activities to a new logistics model involving organ repair centers where DBD and DCD organs can be repaired and possibly optimized during perfusion or optimized preservation. This new logistic may relaunch the organ transplant field, encountering the unmet clinical need of the many patients not receiving an organ or receiving an organ late when outcomes may be less enthusiastic. The availability of a new device that allows non-ischemic preservation of the heart through cold hypothermic blood perfusion, significantly reducing the risk of PGD (risk ratio 0.39) and adverse outcomes (5) promises to revolutionize the field further. If EVMP also expands the technologic armamentarium to pediatric donors and recipients, more patients will be transplanted whose needs are currently not encountered (6).
